# Augmented and Mixed Reality Interventions in People with Multiple Sclerosis: A Systematic Review

**DOI:** 10.3390/brainsci15121292

**Published:** 2025-11-30

**Authors:** María Fernández-Cañas, Roberto Cano-de-la-Cuerda, Selena Marcos-Antón, Ana Onate-Figuérez

**Affiliations:** 1 International Doctorate School, Rey Juan Carlos University, 28922 Alcorcón, Spain; 2 Department of Physical Therapy, Occupational Therapy, Physical Medicine and Rehabilitation, Faculty of Health Sciences, Rey Juan Carlos University, 28922 Alcorcón, Spain; 3 Faculty of Health Sciences, Universidad Villanueva, C. de la Costa Brava, 2, Fuencarral-El Pardo, 28034 Madrid, Spain; 4 Department of Nursing, Physical Therapy, and Occupational Therapy, Castilla-La Mancha University, 45071 Toledo, Spain; 5 Grupo de Investigación en Fisioterapia Toledo (GIFTO), Castilla-La Mancha University, 45071 Toledo, Spain

**Keywords:** multiple sclerosis, neurorehabilitation, mixed reality, augmented reality, extended reality, immersive technologies, rehabilitation outcomes

## Abstract

**Background:** In recent years, extended reality has gained traction in people with multiple sclerosis (MS) for their ability to deliver engaging, task-specific, and multisensory therapeutic experiences. **Aim:** This systematic review investigates the application of Mixed Reality (MR) and Augmented Reality (AR) technologies in neurorehabilitation for individuals with MS. **Method:** A comprehensive systematic review was conducted across seven databases and seven eligible studies were identified involving MR/AR interventions targeting motor and cognitive functions, in accordance with the Preferred Reporting Items for Systematic reviews and Meta-Analyses (PRISMA). The review protocol was prospectively registered in the International Prospective Register of Systematic Reviews (PROSPERO). Data extraction was performed independently by the two reviewers and discrepancies were resolved by consensus or consultation with a third reviewer. Participants were predominantly diagnosed with relapsing-remitting MS and presented mild to moderate disability. Technologies ranged from head-mounted displays to home-based AR platforms, with interventions addressing gait, upper-limb coordination, and dual-task performance. Outcome measures were mapped to the ICF framework, encompassing body function, activity, participation, and contextual factors. **Results:** Findings suggest short-term improvements in gait parameters, grip strength, and motor coordination, with enhanced engagement and usability reported. Methodological quality was moderate, with small sample sizes and heterogeneous protocols limiting generalizability. Risk of bias varied across study designs. Despite promising results, further research is needed to validate long-term efficacy, optimize cognitive load, and standardize intervention protocols. **Conclusions:** MR and AR may serve as effective complements to conventional and VR-based rehabilitation, particularly in personalized, task-oriented training for MS populations.

## 1. Introduction

Neurological disorders often result in long-term impairments that significantly affect patients’ functional independence and quality of life. Among these, multiple sclerosis (MS) stands out as a chronic, demyelinating disease of the central nervous system characterized by heterogeneous symptoms, including motor dysfunction, sensory impairments, and cognitive deficits. Effective neurorehabilitation strategies are therefore essential to mitigate disability progression and promote neural plasticity in individuals with MS [1].

Extended Reality (XR) refers to a spectrum of immersive technologies, including Virtual Reality (VR), Augmented Reality (AR), and Mixed Reality (MR), that enable the integration of digital content into real or virtual environments. In the medical field, XR facilitates enhanced visualization, simulation, and interaction for purposes such as surgical planning, medical education, rehabilitation, and patient engagement, improving both clinical outcomes and training efficiency [2].

In recent years, VR has gained traction in MS neurorehabilitation for its ability to deliver engaging, task-specific, and multisensory therapeutic experiences [3,4,5,6,7,8,9,10,11]. This technology has demonstrated positive outcomes in improving motor performance, balance, and adherence to rehabilitation programs across various neurological populations, including individuals with MS [3,4,5]. VR-based interventions have shown significant efficacy in enhancing functional balance, postural control, and reducing fear of falling in people with MS [10]. However, MR which integrates both real and virtual environments in real time and with the possibility to interact directly both in the real and virtual world—and AR, that overlays digital information—such as images, sounds, or interactive data—onto the real physical environment in real time offers unique advantages by enabling context-aware, interactive, and adaptive therapeutic scenarios that could further enhance rehabilitation outcomes [2].

MR and AR systems in the neurorehabilitation field can deliver dynamic feedback, increase ecological validity, and support multimodal input, making them promising tools for neurorehabilitation. On the other hand, they have been used to enhance therapeutic exercises by providing visual or auditory stimuli, guiding movements, and increasing patient engagement, thereby supporting functional recovery through enriched, task-oriented training experiences.

Despite its potential, the application of MR and AR in neurorehabilitation remains an emerging field in people with MS. While numerous studies have explored VR interventions in MS rehabilitation [5,6], there is a notable paucity of research specifically addressing the role and effectiveness of MR and AR in this population [6]. In addition, the increasing integration of these technologies in neurorehabilitation has been accompanied by a growing concern regarding their psychological and physiological side effects. Among the most pressing of these is the phenomenon often referred to as cybersickness, encompassing symptoms such as disorientation, nausea, anxiety, sensory mismatch, and emotional unease during or after immersive XR exposure [12,13,14].

Given the distinct capabilities of MR and AR to support personalized and engaging rehabilitation experiences, a systematic examination of the existing literature is warranted to identify current applications, technological advancements, and clinical outcomes associated with MR and AR-based interventions for people with MS.

## 2. Objective

The aim of this systematic review is to identify, categorize, and critically appraise all existing studies that report the use of MR and AR technologies in the neurorehabilitation of individuals with MS. Secondary objectives include the analysis of methodological quality, levels of evidence and degrees of recommendation and, finally, the risk of bias.

## 3. Methods

### 3.1. Design

A systematic review was conducted in accordance with the Preferred Reporting Items for Systematic reviews and Meta-Analyses (PRISMA) (Supplementary Material Table S1) [15]. The review protocol was prospectively registered in the International Prospective Register of Systematic Reviews (PROSPERO) under registration number CRD420251110808.

This systematic review will be guided by a structured clinical question formulated using the PICO framework (Population, Intervention, Comparison, Outcome). The PICO model provides a systematic approach for defining the scope and objectives of the review, ensuring relevance to clinical practice and research. Specifically, the review will focus on individuals with MS (Population), the use of MR and AR in neurorehabilitation (Intervention), compared to conventional treatments, virtual reality, or no intervention (Comparison), with outcomes related to any body function and structure, activity or participation domains within the International Classification of Functioning, Disability and Health, ICF (Outcome).

### 3.2. Literature Search in Databases

A comprehensive literature search was conducted by two reviewers (AOF and RCC) across these electronic databases: PubMed, Scopus, Web of Science, PEDro, Cochrane Library, CINAHL and Google Scholar, covering all publications from database inception until 30 October 2025. Additional relevant studies will be identified (if applicable) through backward citation tracking of the selected articles.

Combinations of keywords (Medical Subject Headings -MeSH- and free terms), including truncation for the different variations of words, were connected by Boolean operators (Table 1):

### 3.3. Study Selection

Studies were eligible if they met all the following criteria: (i) people with MS with no age limit; (ii) studies including the use of MR and/or AR regardless of the source language; (iii) to provide any outcome measures as an effect of the intervention; (iv) to apply MR and/or AR technology in a neurorehabilitation context. (v) Studies were included if they involved MR and/or AR, or interventions that incorporated any interaction with the real environment. Interventions that partially reintegrated real-world visual information or maintained awareness of the physical environment were considered eligible, as this positions them within the XR continuum.

This systematic review excluded articles according to the following exclusion criteria: (i) studies published as study protocols, theoretical papers and clinical trial registration; (ii) studies that use other technologies; (iii) systematic or non-systematic reviews. (iv) VR studies were excluded if the intervention fully substituted the real environment, with no integration of real-world feedback during the task.

### 3.4. Data Collection

Data extraction was performed independently by the two reviewers using a standardized and piloted extraction form to collect data about: authors, country, sample, technology employed, comparison, dosage, outcome measures and main findings.

Due to the anticipated heterogeneity in the study designs and aims, a meta-analysis was not initially proposed. Instead, results were synthesized narratively and presented in tabular form.

All retrieved records were imported into a reference management software, and duplicates were removed. Two independent reviewers (AOF and RCC) screened titles and abstracts against the eligibility criteria. Full texts of potentially relevant articles were obtained and assessed independently by the same reviewers. Articles retrieved through reference lists were also considered. Discrepancies were resolved by consensus or consultation with a third reviewer (MFC).

### 3.5. Methodological Quality of Selected Studies and Risk of Bias

Generally, systematic reviews have focused exclusively on randomized clinical trials (RCTs), thus discarding observational studies and diagnostic studies due in part to difficulties in their evaluation of methodological quality. However, in many areas of health care there is a lack of quality RCTs, and to guarantee a critical and objective evaluation of studies not included in this category, proven tools are available, such as the Quality Index of Downs & Black [16]. Although it is true that the design of cohort or case-control RCTs presents fundamental differences, in all of them it is necessary to analyse the characteristics of the intervention, the confounding factors and the results. In the present systematic review, considering the type of studies included, the Quality Index of the Downs & Black tool was used to evaluate their methodological quality. The tool consists of 27 items that assess: content quality (10 items), external validity (3 items), internal validity and bias (7 items) and confounding factors (6 items), in addition to weighting statistical power (1 item). Higher scores indicate higher methodological quality, with a maximum possible score of 27 points.

Additionally, the articles were classified according to the levels of evidence and grades of recommendation for diagnosis studies established by the Oxford Center for Evidence-Based Medicine [17].

To assess methodological quality and risk of bias, three established and internationally validated instruments were selected, each chosen according to the study design to ensure that the assessment criteria matched the specific methodological characteristics and sources of potential bias inherent to each type of research. Specifically, randomized controlled trials, non-randomized intervention studies, and diagnostic accuracy studies present different risks, structures and reporting requirements; therefore, applying a single tool to all designs could lead to inadequate or misleading assessments. For this reason, the following instruments were employed:

(a) Randomized controlled trials—RoB 2 [18]

Randomized clinical trials were evaluated using the RoB 2 tool, selected because it is the standard instrument recommended by the Cochrane Handbook for Systematic Reviews of Interventions for assessing internal validity in randomized studies. RoB 2 evaluates six domains (selection bias, performance bias, detection bias, attrition bias, reporting bias, and other methodological risks) through structured signalling questions that allow classification of studies into low, high, or unclear risk. Two independent reviewers performed the assessment, with disagreements resolved by discussion with a third reviewer. RoB 2 was also used to generate visual summary diagrams [19,20].

(b) Non-randomized intervention studies—ROBINS-I V2

Non-randomized studies were assessed using ROBINS-I V2 (Risk Of Bias In Non-randomized Studies of Interventions), chosen because it is specifically designed to evaluate internal validity in observational or quasi-experimental designs in which randomization is not feasible. It assesses seven domains, including confounding, classification of interventions, participant selection, deviations from intended interventions, missing data, measurement of outcomes, and selective reporting. This tool applies structured decision algorithms to convert responses into domain-level and overall risk-of-bias ratings (low, moderate, serious, or critical). Its use ensures methodological consistency and comparability across non-randomized designs [19,21].

(c) Diagnostic accuracy studies—QUADAS-2

Studies evaluating diagnostic performance were assessed using QUADAS-2, selected because it is the most widely accepted instrument for evaluating bias and applicability in diagnostic accuracy research. QUADAS-2 scores four domains (patient selection, index test, reference standard, and flow/timing) as low, high or unclear risk of bias, following structured guiding questions. Applicability concerns are also rated for patient selection, index test and reference test, allowing transparent appraisal of whether the study procedures align with the aims of this review [22].

In summary, all studies were assessed using the tool that corresponds to their methodological design, ensuring that each study type was evaluated with criteria that capture its specific strengths and potential biases. This structured approach guarantees methodological coherence and improves the comparability and interpretability of the risk-of-bias assessment across the included literature.

## 4. Results

**Figure 1:**
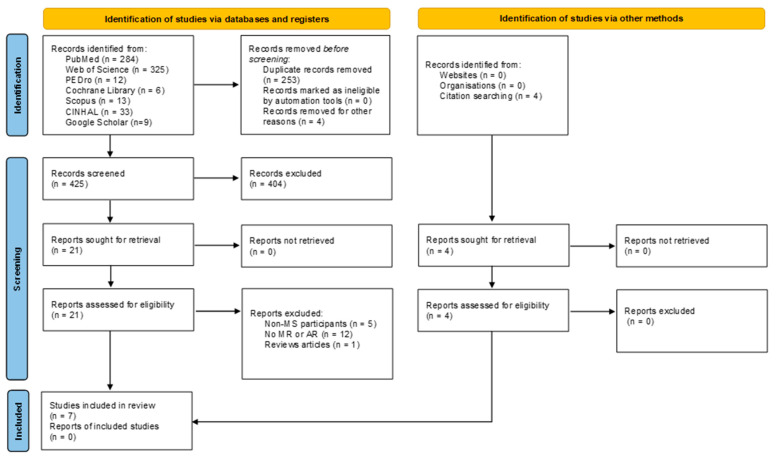
Flow chart.

The identification of articles in the different databases and via other methods and their selection process are detailed in Figure 1. The initial search found 686 articles. After eliminating duplicates and those that did not meet the inclusion criteria, a total of 7 articles were obtained in the present systematic review [23,24,25,26,27,28,29]. The characteristics of the articles are shown in Table 2.

### 4.1. Sample Characteristics

According to the studies included, EM participants presented mild to moderate disability levels, typically reflected in EDSS scores below 6.0. Sample sizes were modest, ranging from 9 to 30 individuals with MS, and most cohorts had a higher proportion of women, consistent with the epidemiological profile of the disease. The mean age of participants varied from early adulthood to late middle age, and disease duration ranged from a few months to approximately ten years, introducing variability in functional baselines that may have influenced outcomes.

### 4.2. Technologies Included and Intervention Characteristics

The technological platforms varied significantly. Early work by Baram & Miller [23,24] employed head-mounted display systems providing closed-loop visual feedback, while more recent studies [25,27,29] leveraged advanced MR systems such as the HoloLens2 integrated with Unity and the MRTK3 toolkit. Others employed home-based AR software (Neuroforma) [27], fNIRS-based cognitive monitoring [25], or treadmill-based AR environments [23,24,25,28]. Intervention durations ranged from single-session exposures [23,24,25,28] to multi-week home-based programs [26], reflecting heterogeneity in dosing protocols. On the other hand, two studies explored the use of MR technologies to assess and improve upper-limb function in people with MS [27,29].

Functional tasks also differed, with some studies focusing on upper-limb fine motor control [26,27,29] and others on gait modulation under various cognitive and environmental conditions [23,24,25,28]. Hernández et al. [25] introduced dual-task paradigms to simulate real-world complexity, while others evaluated isolated sensorimotor metrics under more controlled conditions [25,26,27,29].

### 4.3. Outcomes Measures

Eighteen distinct outcome measures were identified across the seven included studies [23,24,25,26,27,28,29], owing to the reporting of multiple metrics in certain investigations. These measures were mapped to the International Classification of Functioning, Disability and Health (ICF) domains of Body Functions, Activities and Participation, Environmental Factors, and Personal Factors [30].

Physiological and impairment-level metrics were mapped to the ICF domain of Body Functions and comprised 67% of all reported outcomes (*n* = 12). These outcomes included gait motor-control precision and consistency [25]; attentional and cognitive demand during walking [25], three-dimensional hand and eye kinematics [29], fine motor function and coordination [26], finger and hand movement speed and amplitude [26], manual grip strength [26], neuroplasticity markers [26], movement smoothness [27], upper-limb movement efficiency [27], motor planning indices [27], eye–hand coordination [27,29], and cardiac-rhythm responses [28].

One outcome measure (6%) was allocated to the ICF domain of Activities and Participation (walking capacity, assessed as gait speed and stride length) [23,24,25,28]. Two measures (11%) were classified under Environmental Factors: HoloLens 2 technical performance [29] and VR/AR usability [28]. Finally, three measures (17%) were categorised as Personal Factors—namely perceived workload [27,28], user experience [28], and technology acceptance/satisfaction [28]—thus highlighting individual-level modifiers of engagement and comfort.

This ICF-guided mapping demonstrates a predominance of organ-level, impairment-focused outcomes, with comparatively fewer measures addressing participation, environmental facilitators, or personal modifiers, thereby underscoring the necessity for more holistic assessment frameworks in future VR-based rehabilitation research.

### 4.4. Main Findings

Across studies, AR/MR-based interventions demonstrated short-term improvements in gait parameters such as stride length and velocity [23,24,25,28], with more pronounced effects in patients with initially lower gait performance. Similarly, improvements in upper limb motor coordination and grip strength were observed after home-based AR training [25]. Advanced MR assessments, such as those by Sabatino et al. [27], enabled fine-grained quantification of oculomotor coordination and motor smoothness, revealing distinct patterns in patients with cerebellar symptoms.

Cognitive-motor interactions were further illuminated by Hernández et al. [25], who showed increased prefrontal activation and impaired gait accuracy under dual-task AR conditions, particularly in MS participants, highlighting the potential cognitive load imposed by complex AR environments. Meanwhile, Winter et al. [28] reported increased motivation and perceived presence under immersive MR conditions, although physiological markers like heart rate remained unaffected.

According to the reviewed studies, no clinically relevant adverse effects associated with the use of virtual, augmented, or mixed reality interventions in individuals with multiple sclerosis were reported. Only Winter et al. [28] explicitly assessed cybersickness using the Simulator Sickness Questionnaire, observing minimal symptoms without clinical significance.

### 4.5. Assessment of Methodological Quality of the Studies and Risk of Bias

The mean score in the Quality Index of the Downs & Black tool was 16,33 points, which indicates a moderate methodological quality of the studies included. The research with the highest score was Winter et al. [28]. The paper with the lowest score was Baram & Miller [23]. The major concerns with the scale were with the items related to internal and external validity, and with the reporting of data related to the recruited samples.

Table 3 shows the levels of evidence and grades of recommendation. Of note, the studies by Baram & Miller [24], Pruszyńska et al. [26] and Winter et al,. 2021 [28] had the highest grade of evidence (1b). The lowest grade of evidence [4] fell on the work of Bucchieri et al. [29]. Baram & Miller [24], Pruszyńska et al. [26] and Winter et al., 2021 [28] had a grade of recommendation of A. Hernández et al. [25], Sabatino et al., 2025 [27] and Bucchieri et al. [29] had a grade of recommendation of C.

**Figure 2:**
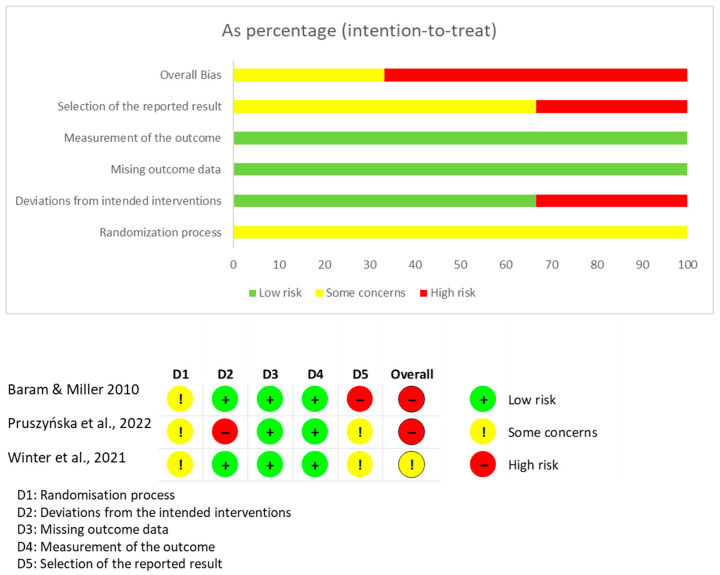
RoB 2 for randomized clinical trials. Sources: Baram & Miller [24], Pruszyńska et al. [26] and Winter et al. [28].

**Figure 3:**
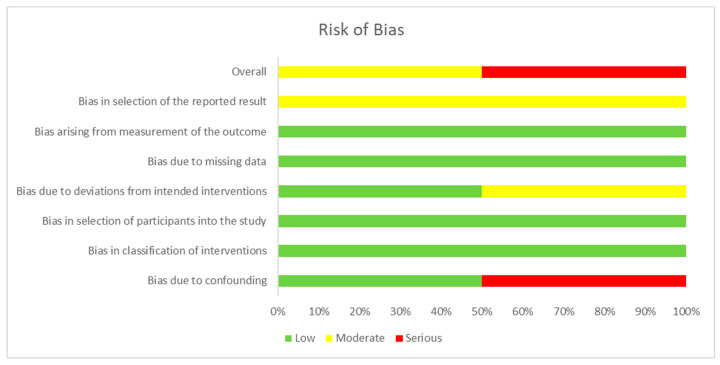

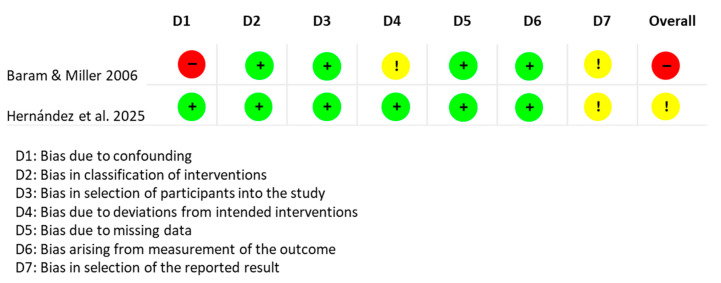
ROBINS-I V2 for non-randomized clinical trials. Sources: Baram & Miller [23] and Hernández et al. [25]

**Figure 4:**
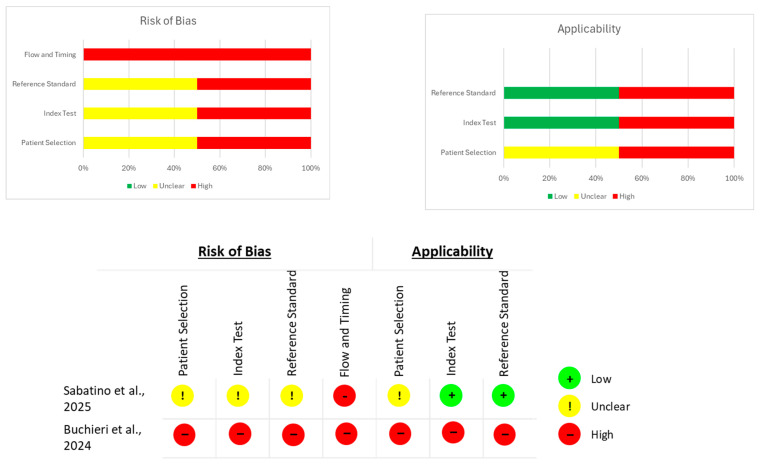
QUADAS-2 evaluation for diagnostic studies. Sources: Sabatino et al. [27] and Buchieri et al. [29].

The assessment of the risk of bias for each type of study is presented in separate figures according to the corresponding tool. Specifically, Figure 2 shows the results obtained using RoB 2, Figure 3 displays the ROBINS-I V2 assessments, and Figure 4 presents the QUADAS-2 evaluation. These figures provide a visual summary of the methodological quality of the included studies.

## 5. Discussion

MR and AR technologies have emerged as promising tools in the field of neurorehabilitation. Across the seven studies included in this review, AR and MR interventions for MS primarily targeted gait modulation and upper-limb motor control through head-mounted displays, treadmill-based AR environments, and home-based AR platforms. Consistent short-term improvements were observed in gait parameters and manual dexterity. Advanced MR systems enabled detailed assessments of oculomotor coordination and motor planning, particularly in patients with cerebellar symptoms. Overall, usability and patient engagement were high, and no clinically relevant adverse effects were reported; only one study explicitly evaluated cybersickness, finding minimal symptoms without clinical significance. Despite these encouraging results, methodological quality was moderate, sample sizes were small, and intervention protocols were heterogeneous, limiting generalizability and preventing conclusions about long-term efficacy. Future research should prioritize standardized protocols, larger cohorts, and follow-up assessments to confirm the therapeutic potential of AR/MR in MS rehabilitation.

### 5.1. Mixed Reality and Augmented Reality in Multiple Sclerosis

The clinical effects of VR-based interventions in people with MS are supported by converging evidence across systematic reviews and meta-analyses. Most studies report improvements in balance, especially static balance, with some demonstrating clinically meaningful changes after higher session volumes [1,10]. Exergaming approaches appear particularly effective, with significant gains in Berg Balance Scale scores [31]. VR has also shown positive effects on fatigue and quality of life, although findings on functional mobility remain inconsistent [32]. While some reviews confirm the superiority of VR over inactivity [33], evidence of advantage over conventional therapy is mixed. Importantly, recent work highlights considerable methodological heterogeneity and stresses the need for standardized protocols to enhance clinical translation [34]. However, to our knowledge, no previous systematic reviews have specifically examined the application of AR and MR technologies in the field of neurorehabilitation. This represents a significant gap in scientific literature, considering the growing interest in these emerging tools and their potential to complement or enhance existing VR-based interventions.

### 5.2. Methodological Quality

Several methodological limitations emerge across studies. First, sample sizes were generally small and often underpowered for subgroup analyses, particularly when comparing phenotypes within MS or across age groups. Second, the short duration of many interventions—often limited to single sessions—restricts conclusions about long-term benefits or neuroplastic adaptations. Moreover, follow-up assessments were infrequently conducted, limiting insight into the persistence of observed effects.

Third, the heterogeneity in technology (hardware and software), dosage, and task complexity complicates cross-study comparisons and the establishment of standardized protocols. While high-tech platforms (e.g., HoloLens2) offer precision and ecological validity, they may be cost-prohibitive or logistically unfeasible in many clinical settings.

Cognitive demands introduced by AR/MR systems also warrant caution. Increased mental workload—especially under dual-task conditions—can be counterproductive for some patients, potentially exacerbating fatigue or interfering with motor control. Lastly, few studies included robust measures of user acceptability or accessibility for individuals with significant cognitive or sensory impairments, except for the study by Winter et al. [28].

### 5.3. Augmented Reality in Other Neurological Diseases and Contexts

A prior scoping review examined the use of VR and AR in biomedical engineering from 2009 to 2023, categorizing 77 studies into four areas: Surgery (including neurosurgery, spine, oral and maxillofacial procedures, and AR-based human–robot interaction), Medical Education (training programs and XR integration in biomedical curricula), Rehabilitation (stroke rehabilitation during COVID-19 and VR interventions in oncology), and AR/VR Systems (technological developments such as millimeter-wave and MIMO systems) [35]. The review highlighted VR and AR as emerging tools in surgery, education, and therapeutic rehabilitation, supported by advances in wireless communication.

Recent research has expanded on their use in neurorehabilitation. A narrative review of VR and AR in post-stroke rehabilitation showed that immersive and gamified interventions can foster neuroplasticity, improve functional recovery, enhance patient engagement, and enable telerehabilitation for underserved populations [36]. This is relevant since, in our systematic review in MS, only Pruszyńska et al. [26] applied AR/MR for telerehabilitation.

In addition, only Pruszyńska et al. [26] targeted upper-limb rehabilitation, indicating the need for further studies on the therapeutic potential of these tools for upper-limb motor deficits in MS, taking advantage of their ecological implementation, user acceptance, and satisfaction.

Research in other populations also supports the effectiveness of AR for gait training. A recent study on AR treadmill-based gait adaptation in community-dwelling post-stroke patients showed meaningful improvements in walking speed, obstacle avoidance, and turning, demonstrating that immersive and cognitively challenging training can boost motor adaptability and functional independence [37]. A systematic review and meta-analysis likewise confirmed the positive effects of AR on rehabilitation outcomes in stroke, improving motor function and daily activities [38]. Overall, these findings show that AR is strengthening traditional rehabilitation and offering evidence-based strategies for motor and cognitive improvement across neurological conditions, consistent with our MS results. Future work should establish protocols to enhance motor performance, patient engagement, and explore telerehabilitation options to expand access for underserved MS populations.

### 5.4. Mixed Reality in Other Neurological Diseases and Contexts

A 2023 scoping review of mixed reality (MR) for acquired brain injury included 26 studies, all in stroke, mainly focused on upper-limb training. Overall evidence quality was low, with a median technology readiness level of 6, reflecting heterogeneity, small samples, and usability issues that limit conclusions about clinical efficacy [39].

Recent MR developments have prioritized feasibility and usability over demonstrating superiority. Foundational work proposed interdisciplinary design principles for MR neurorehabilitation, such as patient-specific task tailoring, feedback loops, and workflow integration [40]. Colomer et al. (2016) evaluated an MR system for upper-limb rehabilitation after chronic stroke, showing feasibility, safety, and preliminary functional gains, supporting its value for repetitive, task-oriented therapy in motivating environments [41]. Likewise, a clinical usability study of Holoreach for trunk and arm control (15 patients, 10 therapists, four sessions each) reported high motivation and perceived benefit, while also noting hardware and software refinement needs; one fainting episode was deemed unrelated to device use [42]. These findings support feasibility rather than comparative effectiveness.

In cerebellar ataxia, two studies showed progression from concept to patient testing. A 2023 proof-of-concept described a HoloLens 2 exergame with therapist-configurable tasks and demonstrated outputs in a healthy subject [43]. A 2024 study with eight patients and eight controls used holographic reach-and-grasp tasks and identified kinematic metrics—time, sway area, trajectory deviation—that distinguished patients and supported a classifier; participants also reported system trust. The authors suggested aligning these metrics with SARA for a potential disease index, though longitudinal evidence is still lacking [44].

Device preference varies by context. In a pediatric study comparing MR and VR headsets in 13 youths with neuromotor disorders, most preferred VR for richer object appearance, whereas therapists favored MR for supporting movement performance; cybersickness was minimal [45]. No comparable studies were found in MS, highlighting the need to examine acceptance of MR versus AR in this population.

Overall, MR in neurological disorders shows promise for precise kinematic assessment, patient-tailored task design, and clinical workflow integration [39,40,41,42,43,44], yet superiority over standard care or VR is unconfirmed. Future studies should include rigorous trials with adequate power, standardized outcomes, transparent adverse-event reporting, and co-design approaches to address hardware and software limitations reported by users [39,42]. Until stronger evidence emerges, MR should be viewed as a feasible and measurement-rich complement rather than a proven superior intervention.

### 5.5. Clinical Implications

The findings of this systematic review suggest that AR and MR technologies hold promising clinical potential for the rehabilitation of individuals with MS. These systems offer interactive and adaptive environments that may enhance patient engagement, motivation, and adherence to therapy. Moreover, their capacity to deliver real-time feedback and integrate multisensory stimuli can support motor and cognitive rehabilitation in a personalized manner. Despite the limited number of studies, early evidence indicates that AR and MR may serve as effective complements to conventional and VR-based interventions, particularly in gait (speed and stride length) and dexterity and manipulative skills. Additionally, AR and MR could be interesting tools as objective and enriched environments. However, standardized protocols and further clinical validation are needed before their widespread implementation in routine neurorehabilitation practice (Table 4).

### 5.6. Limitations

There are different limitations in this review that should be highlighted. Given the heterogeneity of the study designs, technologies used and dosages, it was impossible to develop a meta-analysis. Both the methodological quality of the studies and the risk of bias were low to moderate, with issues to be improved in future study designs as patient selection, confounding variables, flow, and timing, so the conclusions of the different studies should be taken with caution. Other limitations that could be highlighted are the low level of recommendation of some of the studies [25,27,29] and the low sample size of most of the studies. Finally, the absence of data on the EDSS score, the forms of presentation of the disease, as well as the years of evolution of the disease do not allow us to extrapolate the results of the investigations to all subjects with MS.

## 6. Conclusions

MR and AR technologies represent promising tools for neurorehabilitation in individuals with MS. The studies reviewed indicate that these immersive, interactive, and adaptive systems can enhance patient engagement, motivation, and adherence to therapy. MR and AR interventions have demonstrated short-term improvements in motor performance, including several gait parameters (gait speed and stepping accuracy) and upper-limb dexterity, while also enabling precise, fine-grained assessment of kinematic and cognitive-motor (dual tasks) interactions.

Despite these encouraging findings, the overall methodological quality of the evidence is low to moderate, sample sizes are small, and intervention protocols are heterogeneous in terms of technology, dosage, and task complexity. Long-term benefits, neuroplastic adaptations, and comparative effectiveness versus conventional or VR-based rehabilitation remain largely untested. Furthermore, cognitive demands, accessibility, and usability considerations require careful attention to optimize clinical applicability.

In conclusion, MR and AR might have the potential to serve as interesting complements to traditional neurorehabilitation approaches, in terms of motor control assessment and as a potential treatment option for both the upper and lower limbs. However, before widespread clinical implementation, rigorous, adequately powered trials with standardized outcome measures, long-term follow-up, and co-designed, patient-centered protocols are necessary to validate their efficacy, safety, and usability in diverse MS populations.

## Figures and Tables

**Table 1:** Combinations of keywords in databases.

Data Base	Search Fields	Search Strategy	Filters
PubMed	MeSH terms + free-text keywords	multiple sclerosis (MeSH) AND rehabilitation (MeSH) AND mixed reality OR augmented reality	Clinical trial, controlled clinical trial, clinical study
Web of Science	Topic	multiple sclerosis AND rehabilitation AND (mixed reality OR augmented reality)	Clinical trial
PEDRo	Free-text keywords	multiple sclerosis, rehabilitation, reality	No filters
Cochrane Library	MeSH terms + free-text keywords	multiple sclerosis AND rehabilitation AND (mixed reality OR augmented reality)	No filters
Scopus	Title, abstract and keywords	multiple sclerosis AND rehabilitation AND (mixed reality OR augmented reality)	No filters
CINHAL	Free-text keywords	multiple sclerosis AND (mixed reality OR augmented reality)	No filters
Google Scholar	Advanced search fields	With all of the words: multiple sclerosis; With the exact phrase: mixed reality, augmented reality; Where my words occur: anywhere in the article	No filters

**Table 2:** Main characteristics of the included studies.

Author/ Year/ Country	Participants (Sample Size/Age, Sex/Disease Duration/EDSS)	Technology Employed	Intervention or Protocol	Dose	Outcome Measures	Results	Downs & Black Scale
Baram & Miller, 2006 [23]/Israel	*n* = 28 Group 1: *n* = 16 MS/40.3 (± 13.5)/56.2% women/8.6 (± 8.1) months/4.4 (± 1.3) Group 2: *n* = 12 healthy control/25.4 (±1.9)/50% women	A closed-loop head-mounted display (HMD) system projecting either a checkerboard floor or transverse lines onto a self-paced treadmill.	Each participant completed 4 walking stages of 10 m at a self-selected comfortable speed. - Baseline (no HMD). - HMD worn, display off. - HMD worn, display on (active Virtual Reality). - Post-Virtual Reality, no HMD after a 10-min rest (short-term residual). A healthy control group underwent the same.	1 session	- Walking speed (m/s) - Stride length (m)	MS with low baseline speed (<0.7 m/s): On-line speed gain: +13.46% Residual speed gain: +24.49% On-line stride gain: +9.78%; residual: +16.32% MS with high baseline speed (>0.7 m/s): On-line speed gain: +1.47% Residual speed gain: +9.09% On-line stride gain: +5.74%; residual: +4.86% Healthy controls: No meaningful improvement.	10/27
Baram & Miller, 2010 [24]/Israel	*n* = 21 Group 1: *n* = 10 MS/39 (±13)/70% women/8 (±5.8) months/3.8 (±1) Group 2: *n* = 11 MS/42.7 (±14)/64% women/8.6 (±8.8) months/4.9 (±1.2)	A closed-loop head-mounted display (HMD) system projecting either a checkerboard floor using a “glide-symmetric” “or transverse lines onto a self-paced treadmill	- Group 1: transverse-line visual cues - Group 2: checkerboard-tile visual cues - Baseline assessment: four 10 m walks without the device (speed and stride averaged). - Training: 20 min of continuous walking with the VR device active, imagining stepping on the lines or tiles. - Rest: 10 min break without the device. - Post-training assessment: four 10 m walks without the device (speed and stride averaged).	1 session	- Walking speed (m/s) - Stride length (m)	Group 1: Speed gain: 7.79% ± 4.24% Stride gain: 7.20% ± 3.92% Group 2: Speed gain: 21.09% ± 18.39% Stride gain: 12.99% ± 11.72% Group 2 vs. group 1: both speed and stride improvements were significantly greater (*p* < 0.05). - Within Group 2, patients with below-median baseline speed improved markedly more (32.61% ± 22.71%) than those above the median (10.52% ± 10.12%; *p* < 0.05).	15/27
Bucchieri et al., 2024 [29]/Italy	*n* = 9 MS Age, sex, disease duration and EDSS: Not reported	Microsoft HoloLens 2 + Mixed Reality Toolkit (MRTK) + PTC Vuforia extension + two Mixed Reality exergames (ROCKapp for transversal pick-and-place; PICKapp for sagittal pick-and-place).	- Order of tasks: 1° ROCKapp then PICKapp - Repetitions: 5 trials × 6 movements (30 total) per application - Session duration: ~45 min	1 session	System performance Frames per second (FPS) over each app session Percentage of missing hand-tracking samples. Data integrity Eye-tracking continuity	FPS: around 50 Hz in both applications; no significant difference between apps (*p* = 0.14) or across the five repetitions of each (*p* > 0.05). Missing hand-tracking samples: ROCKapp = 2.98%; PICKapp = 13.9%. Eye-tracking fully consistent (0% data loss).	16/27
Hernández et al., 2025 [25]/United States	*n* = 32 Group 1: *n* = 10 healthy young adults/21.9 (±3.4)/50% women Group 2: *n* = 12 healthy older adults/63.1(±4.4)/75% women Group 3: *n* = 10 MS/56.2 (±5.1)/80% women/disease duration not reported/range: 1–6; median = 3.75	C-Mill self-paced instrumented treadmill (Motekforce Link)—AR targets and obstacles were presented on the treadmill belt surface.	- Walking tasks: • Cued walking with visual targets • Obstacle walking with unexpected cues • Dual-task versions with backward alphabet recitation - Pseudorandomized trials with prior practice	1 session	- Stride velocity - Target hit rate - Footfall placement bias and variance (ML error and ML error SD) - Prefrontal cortex oxygenated hemoglobin (HbO) levels	- Stride velocity: Decreased in dual-task conditions for MS (*p* < 0.05) and older adults (*p* < 0.01). - Target hit rate: Lower in obstacle walking, especially in older adults (*p* < 0.001). - Footfall placement bias and variance: MS and older adults showed greater bias and variability (*p* < 0.05). - Prefrontal cortex HbO levels: Increased in dual-task and obstacle conditions for MS and older adults (*p* < 0.05).	18/27
Pruszyńska et al., 2022 [26]/Poland	*n* = 30 Group 1: *n* = 15 relapsing-remitting MS/38.3 (±7.6)/73% women/9.9 (±5.4) years/EDDS not reported Group 2: *n* = 15 relapsing-remitting MS/41.4 (±4.6)/73% women/9.6 (±4.3) years/EDDS not reported	Neuroforma™ Augmented Reality system on computer with standard webcam, which overlays virtual objects and tracks hand movements for home-based upper-limb exercises.	Group 1 (AR intervention): - 8 upper limb exercises (bilateral and unilateral) - Performed seated for safety - Visual and audio feedback during tasks Group 2 (Control): They were advised to perform independent home exercises using both hands in their daily activities.	Group 1: - 4-week training cycle - 5 sessions per week - 40–45 min per session	- 9-hole peg test (9-HPT) - Ball-pulling test - Handgrip strength (dynamometer) - Plasma levels of brain-derived neurotrophic factor (BDNF) and platelet-derived growth factor (PDGF)	- No statistically significant differences were found between groups (*p* > 0.05). - Group 1: demonstrated significant improvements (*p* < 0.05): Manual dexterity (9-HPT), movement speed (ball-pulling test) and handgrip strength in both hands. No significant improvements were observed in Group 2 with respect to these outcomes - No significant changes in BDNF or PDGF levels for any group (*p* > 0.05)	18/27
Sabatino et al., 2025 [27]/Italy	*n* = 21 Group 1: *n* = 9 MS/43.2 ± (13.7)/56% women/disease duration not reported/3.9 (±4) Group 2: *n* = 12 healthy control/42.1 (± 8.1)/67% women	Microsoft HoloLens 2 + ROCKapp Mixed Reality application for hand-tracking and eye-tracking during a pick-and-place task in MS patients.	Participants seated: pick-and-place 30 movements, 5 trials × 6 directions. With their hand resting on the table, they grasped a bottle at one of the holographic positions (North, South, East, or West), moved it in a straight line to another position, released it (triggering a virtual rocket), and then returned their hand to the table. The order of S, E and W moves is randomized; N always serves as the “home” position you return to for SN, EN and WN trials.	1 session	Spatial Arc Length (SPARC). Number of Velocity Peaks (NVP). Movement Time (MT) Symmetry. Kurtosis. Number of Zero Crossing Points (N0C). NASA-TLX Questionnaire	The subgroup of MS participants without cerebellar symptoms showed worse values in SPARC, NVP, MT, kurtosis, N0C, and symmetry (*p* < 0.05). Each non-tremor MS patient (S1, S4, S6, S7) was compared directly against the healthy control group: S1: NVP, MT, symmetry, N0C; S4: MT, kurtosis; S6: SPARC; S7: NVP, MT, N0C (*p* > 0.05). Only the temporal demand subscale of the NASA-TLX differed significantly between groups (*p* ≈ 0.012)	18/27
Winter et al., 2021 [28]/Germany	*n* = 50 Group 1: *n* = 14 (MS 10; stroke 4)/MS 52.9 ± (7.6); stroke 52.9 (±8.2)/MS 70% women; stroke 75% women/EM 18.8 (± 10.1) months; stroke 7.5 (±5.3) months/3.9 (±4)/<6 Group 2: *n* = 36 healthy control/22 ± (3.7)/72% women	Immersive Virtual Reality (HTC Vive Head-Mounted Display (HMD) + foot trackers); semi-immersive VR (55″ monitor); conventional treadmill Augmented reality treadmill: - Virtual shoes projected at the position of the real feet. - Safety features: when participants look down, a live camera image of the real environment is overlaid onto the virtual scene, and green or red arrows appear as warning signals if the user moves too far forward or backward on the treadmill, respectively.	Within-subject design: three treadmill conditions (no VR/monitor VR/immersive VR), each ~7.5 min; speed self-adjusted	Three 7.5 min runs; healthy in one day; patients over two days	- Walking speed (m/s) Heart rate Borg Rating of Perceived Exertion (RPE) NASA-TLX (Raw Task Load Index) Mood (0–10) Motivation (0–10) Sense of time (0–10) VR-Specific Questions (0–10) Presence (IPQ) Intrinsic Motivation Inventory (IMI) Simulator Sickness Questionnaire (SSQ) Equipment & Display Questionnaire (EDQ) System Usability Scale (SUS) User Preference	For both groups, the walking speed in the HMD condition was higher than in treadmill training without VR and in the monitor condition. Healthy participants reported a higher motivation after the HMD condition as compared with the other conditions. Importantly, no side effects in the sense of simulator sickness occurred and usability ratings were high. No increases in heart rate were observed following the VR conditions. Presence ratings were higher for the HMD condition compared with the monitor condition for both user groups. Most of the healthy study participants (89%) and patients (71%) preferred the HMD-based training among the three conditions and most patients could imagine using it more frequently.	19/27

MS: multiple sclerosis; EDDS: Expanded Disability Status Scale.

**Table 3:** Levels of evidence and grades of recommendation.

Study	Level of Evidence	Grade of Recommendation
Baram & Miller 2006 [23]	2b	B
Baram & Miller 2010 [24]	1b	A
Hernández et al., 2025 [25]	3b	C
Bucchieri et al., 2024 [29]	4	C
Pruszyńska et al., 2022 [26]	1b	A
Sabatino et al., 2025 [27]	3b	C
Winter et al., 2021 [28]	1b	A

**Table 4:** SWOT Analysis: Use of Augmented and Mixed Reality in Multiple Sclerosis Rehabilitation and Assessment.

Strengths Innovative Functional Assessment: Mixed and augmented reality systems offer precise, real-time tracking of motor and cognitive performance, as demonstrated in Sabatino et al., 2025 [27] and Baram & Miller, 2010 [24]. These technologies enable quantification of fine motor skills and oculomotor coordination with high ecological validity. Short-term Clinical Benefits: Multiple studies (e.g., Baram & Miller, 2006 [23]; Pruszyńska et al., 2022 [26]) report improvements in gait parameters, coordination, and upper-limb function after AR/MR interventions, even in short durations. Increased Patient Engagement: Immersive environments promote higher motivation and perceived enjoyment during training (Winter et al., 2021 [28]), which could enhance adherence to rehabilitation protocols. Ecological Validity and Dual-Task Feasibility: AR/MR allows for dual-task paradigms that mimic real-world complexity (Hernández et al., 2025 [25]), offering better insights into daily functioning challenges in MS.	Weaknesses Small Sample Sizes and Heterogeneous Designs: All six studies suffer from limited statistical power and high variability in methodology, making cross-study comparisons difficult and generalizability low. Lack of Long-Term Data: No study has yet evaluated the long-term retention of benefits or neuroplastic changes following AR/MR-based interventions. Limited Standardization: There is no standardized AR/MR protocol for MS rehabilitation. Technologies, outcome metrics, and intervention durations vary widely (e.g., single session vs. multi-week programs). Cognitive Load Risks: AR/MR environments may increase cognitive demand, particularly in dual-task settings (Hernández et al., 2025 [25]), which could overwhelm patients with cognitive deficits or fatigue.
Opportunities Development of Tailored Interventions: With further refinement, AR/MR could support personalized rehabilitation protocols based on patient-specific motor or cognitive deficits, including phenotype-specific features like cerebellar dysfunction (Sabatino et al., 2025 [27]). Home-Based Monitoring and Training: Tools like Neuroforma (Pruszyńska et al., 2022 [26]) show the feasibility of home-based AR, offering scalable and remote therapy options that can support continuity of care. Integration with Neuroimaging and Biometrics: Combining MR environments with neurophysiological monitoring (e.g., fNIRS, EEG) as in Hernández et al., 2025 [25] can deepen understanding of brain-behavior interactions in MS. Expansion into Cognitive Rehabilitation: Given the dual-task sensitivity of these systems, they could also support cognitive training in addition to motor rehabilitation.	Threats Technological Barriers and Cost: High-end AR/MR devices (e.g., HoloLens2) are expensive and may not be accessible to most clinical centers or patients. Digital Inequality and Usability Challenges: Older or cognitively impaired individuals may face difficulties using immersive systems without significant support or training. Lack of Clinical Guidelines: The absence of validated protocols and official guidelines may slow the clinical adoption of these technologies for MS care. Potential Overreliance on Visual Feedback: As shown in early studies (Baram & Miller, 2006 [23]), some improvements may result from compensatory strategies rather than true motor learning, limiting long-term efficacy.

## Data Availability

No new data were created or analyzed in this study. Data sharing is not applicable to this article.

## Supplementary Materials

The following supporting information can be downloaded at: https://www.mdpi.com/article/10.3390/brainsci15121292/s1, Table S1: PRISMA 2020 Checklist. Reference [46] is cited in the Supplementary Materials.
